# Evaluating AI Performance in Systematic Literature Reviews for HEOR: A Case Study

**DOI:** 10.36469/001c.165173

**Published:** 2026-08-07

**Authors:** Jing Wang-Silvanto, Mansee Jajoo, He Guo, Rishi Ohri, Judith Nelissen, Carolina Casañas i Comabella

**Affiliations:** 1 Astellas Pharma A/S, filial I Finland, Espoo; Observational Health Data Sciences and Informatics (OHDSI), Finland; 2 Astellas Pharma, Astellas Pharma Global Development Inc. Northbrook, Illinois, USA; 3 Astellas Pharma Europe BV, Leiden, The Netherlands; 4 PPD Evidera Health Economics & Market Access, Thermo Fisher Scientific, London, UK

**Keywords:** artificial intelligence, machine learning, systematic literature reviews, large language models, automation tools, evidence synthesis

## Abstract

**Background:**

Systematic literature reviews (SLRs) are central to evidence generation in health economics and outcomes research, but traditional SLR workflows are labor-intensive. Artificial intelligence (AI) is increasingly being explored to support evidence synthesis tasks.

**Objectives:**

To describe methods used to assess AI performance in screening and data extraction in SLRs, and to provide recommendations on how to ensure appropriate use of AI in literature reviews.

**Methods:**

We conducted an AI-assisted SLR that mirrored a traditional SLR performed by humans only and assessed the performance of the AI tools employed. AI was used to screen titles/abstracts, screen full texts, and conduct data extraction. Using the traditional SLR as the benchmark, AI performance for title/abstract screening was evaluated in terms of accuracy, recall, and precision at predefined screening milestones, followed by the accuracy assessment of full-text screening by AI. Data items extracted by AI were verified by humans and categorized as correct, incomplete, missing, incorrect, or requiring human checks. The average accuracy rate calculated on the basis of correct data items extracted was used as an indicator of AI performance in data extraction.

**Results:**

During title/abstract screening, accuracy and recall remained high but precision was low, increasing false positives. Full-text screening identified a minority of studies included in the traditional SLR. Mean extraction accuracy was 72.93% (range, 57.69%-88.46%). No data were hallucinated by the AI model used.

## INTRODUCTION

Systematic literature reviews (SLRs) are a cornerstone of evidence generation in health economics and outcomes research (HEOR). SLRs use a replicable and transparent approach to identifying, appraising, and synthesizing the data required by regulators, payers, and other stakeholders in HEOR and are regarded as the gold standard evidence synthesis method.^1–3^

The methods traditionally used in SLRs are labor-intensive, time-consuming, and costly, especially conducting double screening and detailed data extraction and validation.^3–5^ This burden is amplified in HEOR, in which decision makers require broad evidence bases and frequent updates to stay current with a growing body of evidence.

Automation tools, text mining, and machine learning (ML) have been used in reviews for over a decade.^6–8^ More recently, other forms of artificial intelligence (AI), particularly large language models (LLMs), have been increasingly explored as a means to accelerate SLRs, especially tasks around literature searches, title and abstract screening, full-text review, and data extraction.^9,10^

LLMs are typically accessed through commercial foundational models/chatbots such as OpenAI’s ChatGPT, Microsoft’s Copilot, or Google’s Gemini. These models generate natural-language text responses to user prompts using advanced pretrained probabilistic algorithms.^11,12^ Although these foundational AI models have been explored for systematic review tasks, they are not designed specifically for evidence synthesis. Consequently, they often lack key features required for review workflows, such as structured and transparent record management, replicable screening decisions, and audit trails. Moreover, concerns have been raised about their accuracy, critical thinking, and the generation of incorrect or unverified information (ie, hallucinations).^11,13,14^

In parallel to generic models, numerous web-based platforms, such as Abstrackr, Rayyan, Covidence, Distiller, and Nested Knowledge (NK), have been developed specifically to support core systematic review activities. These platforms provide structured interfaces for generating and managing searches, importing and deduplicating records, conducting title/abstract and full-text screening, extracting data, and documenting decisions.^15,16^ In recent years, many of these platforms have incorporated ML and other AI-based functionalities aimed at reducing manual workload and accelerating execution. Examples include active learning for citation screening, prioritization algorithms, and semiautomated extraction modules. However, the functionality and the extent to which they use LLMs differs considerably across tools.^15–17^

Platforms such as NK and DistillerSR, for example, offer integrated search connections to key literature databases such as Medline and Embase, respectively, in addition to automated record import, deduplication, and classifier training. Some of these capabilities are delivered through connections to LLM-based application programming interfaces, thus enabling more flexible text handling within otherwise structured review workflows.^9^ By contrast, other commonly used platforms offer only certain AI capabilities, limited to ML-based tools. For example, Abstrackr and Rayyan use ML for title and abstract screening; Covidence, which is endorsed by Cochrane, uses ML and automation for screening, including its validated RCT Classifier, which is trained to identify abstracts that report randomized controlled trials.^9,18,19^ Notably, most of these tools focus their AI screening capabilities on titles and abstracts. However, a recent publication presented the agentic AI platform A4SLR, which includes a module on full-text screening within a full AI workflow to conduct reviews.^20^

Despite these promising advances, several methodological and practical challenges persist and in some cases limit the adoption of AI in SLRs. Some of these challenges apply to generic AI models (eg, ChatGPT, Claude, Copilot) and consist of the lack of review-specific capabilities, limited replicability and reproducibility, lack of robust validation studies, and differences regarding capabilities, user interface, and risk of hallucinations.^10,13^

Other challenges apply not only to generic AI tools but also to review-specific AI platforms. The limited capabilities of most review-specific models mean they cannot be used reliably for key SLR tasks such as full-text screening, risk-of-bias assessment, or report writing. In addition, a key challenge is that the rapid development of AI outpaces rigorous evaluation^11,13,14^ and hinders reproducibility and replicability. For example, the uncertainty about the frequency or availability of new AI models, including updates, along with the lack of or limited transparency from commercial AI platforms, can substantially impact the accuracy and reliability of the information they provide and may require re-evaluation of tools within the same project.^9,14^

Finally, integrating AI systems into review workflows requires a degree of AI literacy from scientific staff to understand algorithm behavior, configure tools appropriately, design efficient prompts, and make informed decisions about how and when to deploy AI. Consequently, the use of AI in reviews necessitates new scientific abilities from reviewers^18^ and modifies traditional SLR workflows.

Despite growing interest in deploying AI within health technology assessment (HTA)–focused SLRs, there is still limited detailed technical guidance on how to design, evaluate, and report AI-assisted reviews. Key open questions include how to assess the validity of AI-assisted processes, reaching consensus on acceptable AI performance for different SLR tasks and types of reviews (eg, systematic, targeted, rapid), and how AI-generated outputs should be reported and presented to stakeholders.^14,21^

Recognizing these gaps, several key organizations have begun to articulate frameworks for responsible AI use in reviews. The United Kingdom’s National Institute for Health and Care Excellence (NICE) issued a position statement in 2024 that expressed caution regarding the use of AI and emphasized the need for transparency, rigor, and human supervision.^1^ Drawing on this work, Canada’s Drug Agency released a position statement that similarly acknowledges the potential benefits of AI but emphasizes transparency, rigor, trustworthiness, and ethical considerations, and encourages judicious use of AI only when appropriate and valuable.^22^

In November 2025, the Responsible Use of AI in Evidence Synthesis (RAISE) initiative proposed a framework for ethical and transparent AI deployment in reviews.^14^ RAISE highlights the importance of documenting AI methods, continuous monitoring, validating outputs, mitigating algorithmic bias, ensuring user training, addressing data protection and copyright requirements, and regularly updating AI systems to ensure effective and ethical use in evidence synthesis.^14^ Cochrane and partner organizations endorse RAISE recommendations. A recent multiorganization position statement reinforces that evidence synthesists remain ultimately responsible for all aspects of their work, including the decision to use AI and automation, and must ensure compliance with legal and ethical requirements. The statement calls for responsible and transparent use supervised by humans and underscores the need for clear public information on tool functionality, validation, and limitations.^14^ Overall, these position statements are consistent with the principles of traditional, non-AI reviews in emphasizing transparency and reproducibility as prerequisites for scientific rigor.^3^

Also in November 2025, the Professional Society for Health Economics and Outcomes Research (ISPOR) working group on AI issued guidelines on reporting the use of AI, specifically LLMs, in HEOR.^23^ Although these recommendations apply broadly to HEOR studies that used LLMs and are not specific to evidence synthesis, the authors illustrated their application using an existing SLR^24^ that leveraged LLM-assisted abstract screening. The resulting ELEVATE-GenAI checklist consists of 10 domains, including accuracy, factuality verification, reproducibility, and robustness,^23^ all of which are key in SLRs. In addition, this working group underscored the need for further research in HEOR to assess the accuracy of LLMs and reach a consensus on appropriate metrics.^23^

Against this background, there is a clear need to establish robust and transparent methods to evaluate AI performance in SLRs that comply with recommendations from emerging guidance and methodological frameworks such as RAISE.^9,11,13,14^ Therefore, this article aims to describe the methods used to evaluate the performance of AI in reviews, specifically in title/abstract screening, full-text screening, and data extraction, and to provide recommendations on appropriate methods to assess the performance of AI in literature reviews.

## METHODS

We conducted an AI-assisted SLR (AIaS) to mirror a traditional SLR conducted using conventional methods (ie, no AI was used).^25^ Both the traditional SLR and the AIaS aimed to synthesize evidence on the economic burden (healthcare resource utilization [HCRU] and costs) of metastatic pancreatic adenocarcinoma. An overview of the methods used in the traditional SLR can be found in **Supplementary Tables S1, S2, and S3**. Each SLR (AIaS and traditional SLR) was conducted by a separate team. The objectives of the AIaS were to explore the performance and potential efficiencies of using AI to assist with the screening and data extraction tasks in SLRs, and to inform future use cases for AI in SLRs in terms of performance and potential time and cost savings.

For this project, the AI capabilities available in the review-specialized online platform NK were applied for title/abstract screening, full-text screening, and data extraction. The results from the AIaS were compared against the traditional SLR and have been reported previously.^25^

The performance of the AI model during the title/abstract and full-text screening phases was measured in the AIaS using three key metrics: accuracy, recall, and precision (**Table 1**).

**Table 1.** Definitions of AI Metrics

**Metric**	**Definition**
Accuracy	Indicates how often the model is correct in classifying records as included or excluded. Higher accuracy is better. Accuracy = (TP + TN)/(TP + TN + FP + FN).
Recall	Reflects the tool’s ability to identify relevant studies. The AI model aimed to achieve high recall (>70%), indicating that the model was less likely to exclude relevant records. Recall (sensitivity) = TP/(TP + FN).
Precision	Measures the proportion of studies flagged by the tool that are relevant, ie, the proportion of relevant abstracts retrieved among all abstracts retrieved. Low precision indicates that the tool is over-inclusive and more likely to include irrelevant records for full-text review; conversely, low precision reduces the risk of missing eligible studies. Precision = TP/(TP + FP).

Abbreviations: AI, artificial intelligence; FN, false negative; FP, false positive; TN, true negative; TP, true positive.

### Use of AI During Title and Abstract Screening

The search results from the traditional SLR were uploaded in plain-text format (ie, an RIS file) to NK, where duplicates were automatically removed.

Title and abstract screening was conducted using an AI-human hybrid model. In this process, NK’s ML model, called Robot Screener, replaced one human reviewer to determine the inclusion or exclusion of each record based on the Population, Interventions, Comparisons, Outcomes, and Study (PICOS) design framework. Therefore, each record was screened by AI and by a human screener. Conflicts were resolved by scientific staff, and the human decisions were considered final.

First, at the start of screening, human reviewers screened a subset of records to train the AI. Following NK’s recommendations, humans reviewed until at least 10 records were included and at least 50 were excluded. Second, the AI model was deployed and set to train automatically after adjudication (ie, final decision) of every 10 additional records. The characteristics of NK’s Robot Screener are presented in **Supplementary Table S4**.

The performance of Robot Screener during title and abstract screening was monitored at key screening milestones (eg, every 10% of records screened). Given that the AI did not have the capability of providing exclusion reasons, this was not evaluated.

### Use of AI During Full-Text Screening

The full text of records included at the title and abstract screening stage were obtained and uploaded to NK as PDFs. At the time this AIaS was conducted, NK did not offer a dedicated AI function designed for full-text screening. A proxy method was developed to evaluate whether AI could identify relevant publications based on the PDFs of articles. Specifically, prompts were created for each PICOS eligibility criterion using NK’s LLM-based Smart Tags, which identified relevant excerpts from the PDF.^26^ The characteristics of NK’s Robot Screener are presented in **Supplementary Table S4**. These prompts were then applied to each PDF using Smart Tags (see **Supplementary Figure S2**).

After this project was completed, the Smart Tags feature evolved into Core Smart Tags and Adaptive Smart Tags, which can be used to recommend key data from full texts to enable their categorization.

Given that Smart Tags cannot assign a screening decision, the output was exported to Excel, and human reviewers made decisions as follows: records were marked as included or excluded in the AIaS when the AI did or did not, respectively, retrieve information for all PICOS elements.

The performance of AI was assessed by comparing the list of full texts included in the AIaS (ie, the AI-retrieved information for each PICOS element) versus the traditional SLR.

### Use of AI During Data Extraction

During data extraction, the Smart Tags feature was used to extract data from the PDFs of included articles (**Supplementary Table S5**). Multilevel question-based prompt trees mirroring the study elements and outcomes from the traditional SLR were built in Smart Tags (**Supplementary Figure S2**). To do this, the hierarchy of prompts designed for full-text screening was expanded and refined by adding specific requests in the prompts for each required variable. The expanded prompts asked the AI to identify defined variables in the article’s PDF, such as population characteristics, study design information, or outcomes, and included relevant terms and scope (**Supplementary Figure S1**).

To evaluate the performance of AI during data extraction, the data extracted by AI were downloaded into Excel (Microsoft) and each data point was checked by one human reviewer against the traditional data extraction table in Excel. Disagreements between the human reviewer and the AI extraction were resolved by a senior systematic reviewer.

Each data element extracted by the AI was categorized as correct, incomplete, missing, incorrect, or if the data extracted required human checks or interpretation (eg, the AI output was confusing; if the AI was unable to infer the data from the text). Data extraction accuracy was calculated by dividing the number of correctly extracted variables in the article by the total number of variables extracted in the article:

Accuracy = No. of Correctly Extracted Variables / Total No. of Variables Extracted

## RESULTS

### AI-Assisted Title and Abstract Screening

The searches from the traditional SLR yielded 1359 records after removing duplicates. Following the screening by humans of an initial set of 52 titles and abstracts, 10 of which were included, Robot Screener was deployed to screen the rest of records (n = 1307).

**Figure 1.**
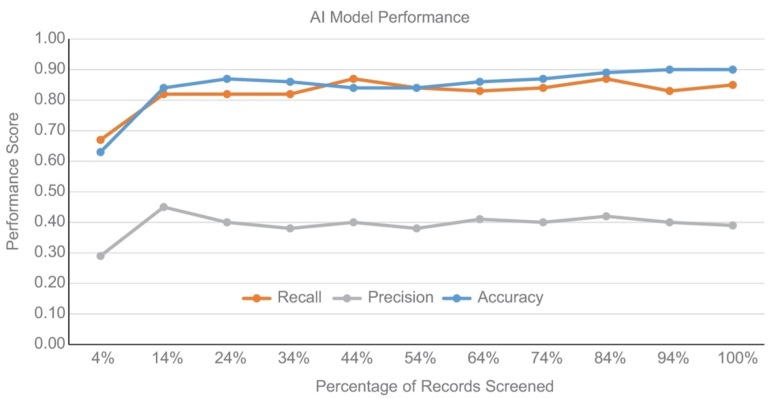
AI Model Performance During Title and Abstract Screening Abbreviation: AI, artificial intelligence.

**Figure 2.**
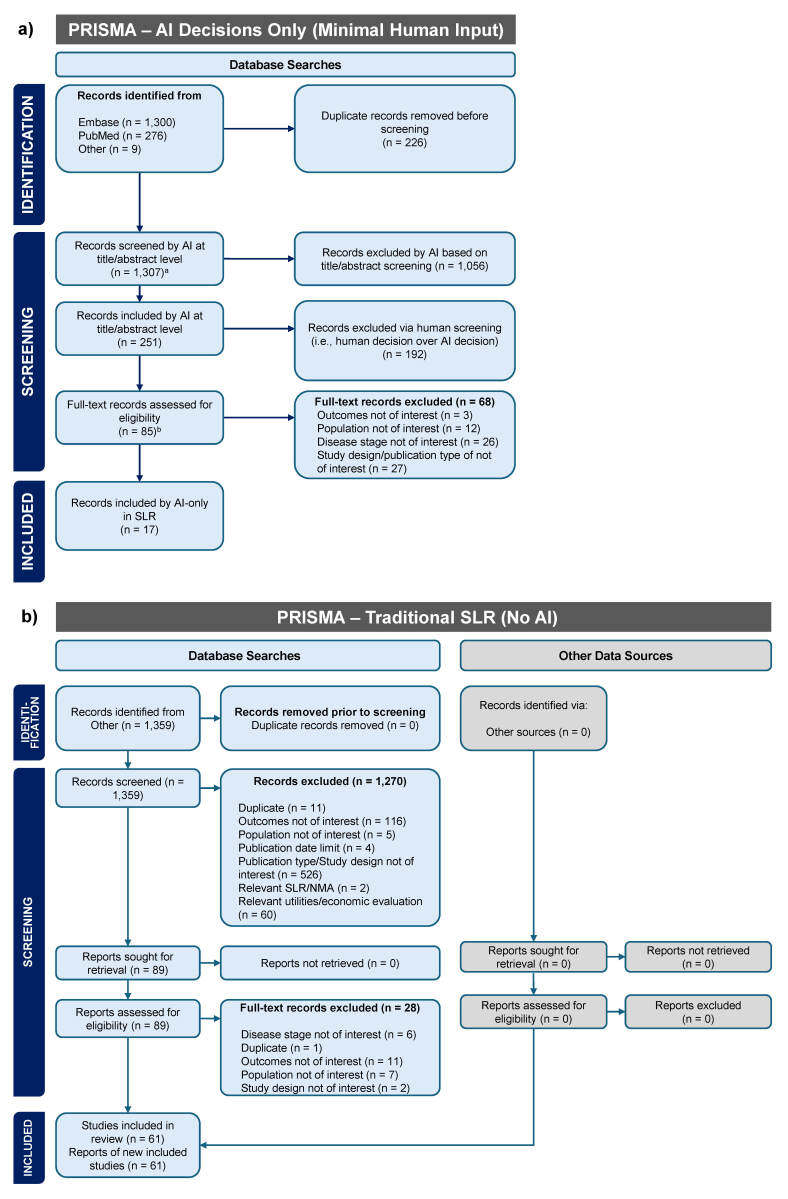
PRISMA Diagrams from AI-Assisted and Traditional Systematic Literature Reviews Abbreviations: AIaS, AI-assisted SLR; SLR, systematic literature review. Panel A presents AI decisions only with minimal human input. Panel B presents the traditional SLR without AI. Panel A excludes the 52 abstracts used as the training set and therefore only screened by humans. Panel A includes records for which the decision was driven by the human screeners (ie, AI excluded but humans included).

After AI screened approximately 25% of 1307 records (ie, 326 abstracts), the AI model achieved an accuracy of 87%, recall of 82%, and precision of 40% (**Figure 1**). The 82% recall indicated that potentially relevant records were less likely to be excluded. Accuracy and recall remained high throughout (range, 85%-90% and 82%-87%, respectively). On the other hand, the low precision (40%) resulted in the inclusion of 251 titles and abstracts by the AI, whereas the traditional SLR had included only 89 (see PRISMA comparison in **Figure 2**).

Once the AI-human hybrid title and abstract screening was completed, scientific staff reviewed the AI decisions. Of the 251 records that the AI deemed relevant, scientific staff excluded 192, resulting in 85 records taken forward to full-text screening in the AIaS. Therefore, there was a discrepancy of 4 abstracts between the AIaS and the traditional SLR because 4 eligible abstracts were not included by the AI at the abstract screening level.

### AI-Assisted Full-Text Screening

The human-driven decisions were applied to determine the number of records at full-text level; thus, 85 publications were taken forward to full-text screening.

**Figure 3.**
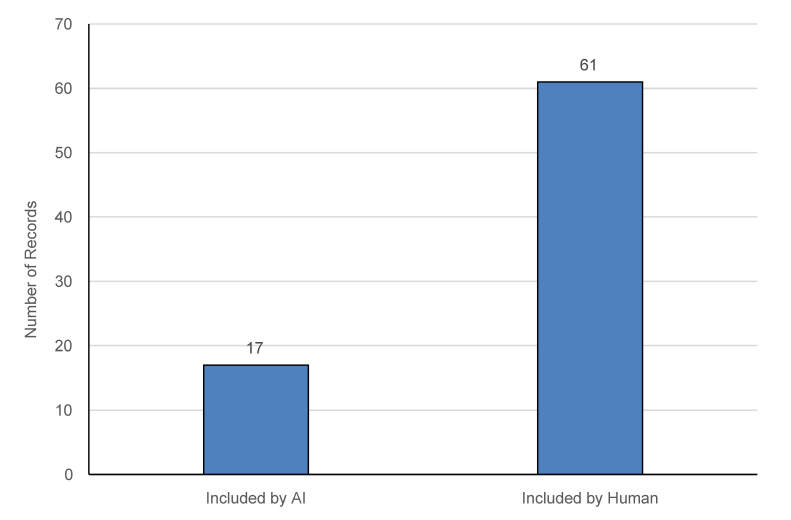
Final Records Included: AI-Assisted vs Traditional Systematic Literature Reviews Abbreviation: AI, artificial intelligence.

Using the proxy method described above for full-text screening, the AI provided information for all PICOS criteria in 20% of articles (17/85), which were therefore considered included by the AI. In contrast, the inclusion rate from the traditional SLR was much higher (68.54% full texts included). When comparing the articles that the AIaS included with the traditional SLR, the AIaS identified only 27.7% (17/61 articles) of relevant publications compared with the traditional SLR (**Figure 3**).

**Figure 4.**
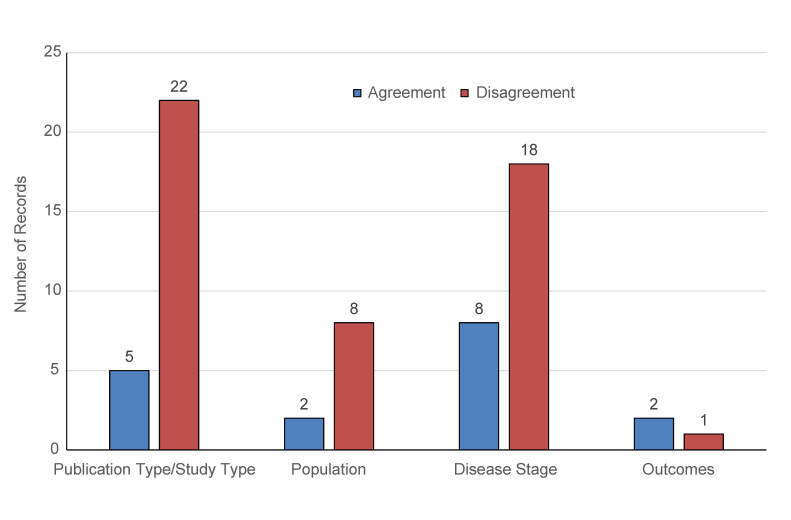
Level of Agreement by PICOS Criterion: AI vs Human Abbreviations: AI, artificial intelligence; PICOS, population, interventions, comparisons, outcomes, and study design.

Comparing the AI performance by PICOS element, the highest proportion of disagreement between human and AI was around publication/study type (81.4%, 22/27 in disagreement) and disease stages (69.2%, 18/27 in disagreement) (**Figure 4**).

### Data Extraction

**Figure 5.**
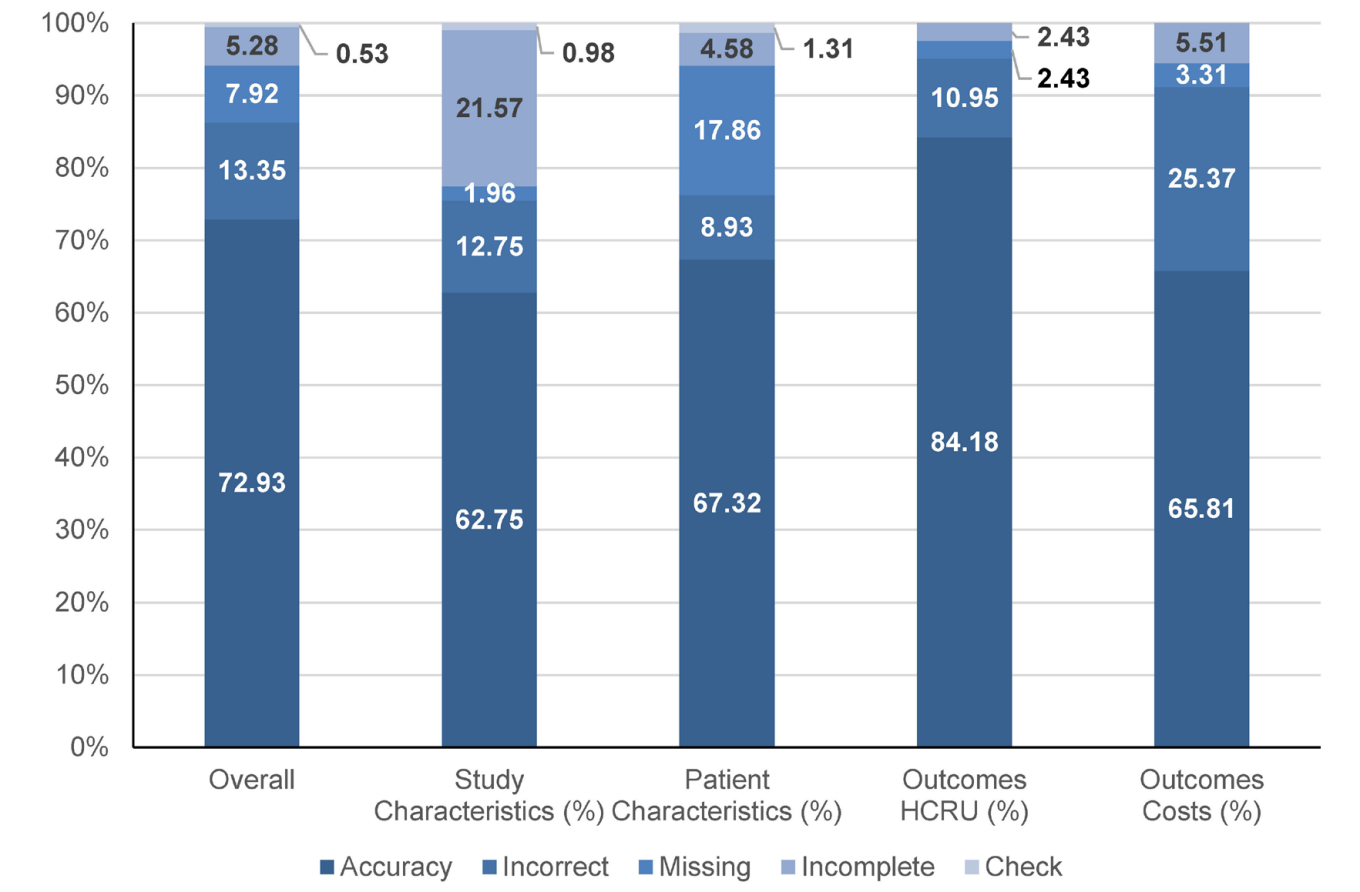
Mean AI Data Extraction Accuracy, Overall and by Type of Information (%) Abbreviations: AI, artificial intelligence; HCRU, healthcare resource utilization.

Across all studies extracted, the mean accuracy for the AI-assisted data extraction was 72.93% (range, 57.69%-88.46%), mean number of incorrect extractions was 13.35% (range, 6.41%-20.51%), mean number of incomplete extractions was 5.28% (range, 1.28%-8.97%), and missing data ranged from 1.28% to 14.10% (mean, 7.92%) (**Figure 5**). None of the individual study extractions achieved 100% accuracy. Incorrect extractions ranged from a mean of 8.93% (range, 3.70%-18.52%) when extracting patient characteristics to 25.37% (range, 0%-56.25%) when extracting costs. Missing extractions ranged from 1.96% (range, 0%-16.17%) to 17.86% (range, 3.70%-33.33%). Extractions that were correct but required human checks were few, ranging from 0.98% (range, 0%-16.67%) to 1.31% (range, 0%-3.70%).

When examined by type of data, accuracy was best when extracting HCRU outcomes (84.18%; range, 58.62%-96.55%) and lowest at study characteristics (62.75%; range, 33.33%-83.33%) and patient characteristics (67.32%; range, 44.40%-85.19%). Incorrect extractions were highest in cost outcomes (25.37%; range, 0%-56.25%), and missing extractions were more common in patient characteristics (17.86%; range, 3.70%-33.33%).

## DISCUSSION

Our AIaS showed that AI can support several review tasks (title and abstract screening, full-text screening, and data extraction) but performs unevenly across stages. We applied key performance metrics including accuracy, recall, and precision.^25^ The NK AI model in this project achieved high accuracy and recall during the title and abstract screening (range, 82%-90%) and sufficient accuracy during data extraction (73%) on average, consistent with previous evidence that ML tools can reliably support abstract screening and data extraction under human supervision.^20,27^ However, low precision resulted in many false positives at the title and abstract screening stage; this limitation has been noted in other models.^28–30^ Full-text screening using a proxy methods identified only a small proportion of relevant studies compared to the traditional review, signaling a need for AI development in this area. Overall, accuracy during data extraction was high at 72.93%; however, mean incorrect extractions were relatively high, at 13.35%. Of note, the quality of AI extractions in the costs outcomes domain presented a low accuracy (65.81%) and a high proportion of incorrect extractions (25.37%). A possible explanation for this may be that costs outcomes are often heterogeneous and inconsistently reported in the literature, requiring interpretations. Consequently, while our findings overall suggest that AI may provide reasonable support for data extraction, some areas require thorough attention and validation by humans.

Human oversight remains a cornerstone of responsible AI use in evidence synthesis, as underscored by recent guidance.^1,14,22^ In our AIaS, human oversight was central to confirming AI-generated decisions and ensuring reliability.^25^ Human supervisors provide critical judgment, domain knowledge, and the ability to contextualize information that current AI models cannot replicate.^1,12,14,20^

As discussed in the introduction, current AI applications present some limitations. For example, AI hallucinations (ie, instances in which AI generates incorrect or unverified information) are a significant concern in evidence synthesis.^11,13,14^ Notably, in our AIaS, NK did not hallucinate content, because the model is trained to extract information only from the PDFs provided, thus minimizing the risk of fabricated data and time spent on validation.^25^ Nonetheless, robust human-led validation processes remain necessary to identify and correct errors, particularly if using generic AI models.

Reproducibility and replicability are at the core of SLR methods.^1,3^ In contrast, LLMs are known to generate nonreplicable outputs because their responses can vary across prompts, sessions, users, or model updates, despite using the same AI tool.^31,32^ In an effort to increase transparency and traceability, the Preferred Reporting Items for Systematic Reviews and Meta-Analyses-Transparent Reporting of AI in Comprehensive Evidence Synthesis (PRISMA-trAIce) checklist has recently been proposed as an extension to the PRISMA 2020 statement to establish a structured way to report the use of AI in evidence synthesis.^21,33^

In addition, there is a lack of formal guidelines for the validation in AI tools in evidence synthesis. Organizations and users should ensure that such AI systems are validated by independent bodies and that the outputs can be accepted by decision-making bodies. Reviewers should also consider the implications of copyright law before implementing AI.^14,23^

Because AI models differ in architecture and training, performance metrics remain fundamental for determining reliability across applications. It is equally important that evidence synthesists using AI tools are AI-literate. Reviewers must understand which AI modality-ML, automation, or LLMs-is appropriate for specific tasks such as title/abstract screening, full-text screening, data extraction, risk-of-bias assessment, or drafting evidence summaries.^9,34,35^ Competence in selecting, supervising, and appraising AI tools is essential to ensure scientific rigor and appropriate AI deployment. Therefore, training and education in AI literacy are vital for maximizing the benefits of AI. Reviewers using AI must not only select the appropriate AI tools for each stage of the review but also understand AI performance metrics to determine how and when to deploy AI. Moreover, they should be able to design efficient prompts for LLM-based tools, which will lead to better AI output and contribute to time and labor efficiencies.

Our project presents some limitations. First, the findings were limited to a single AI platform. Other AI tools may produce different results. Second, as noted in the methods described, NK did not have a native full-text AI functionality, and therefore an adapted method was used. Consequently, the findings on the quality of full-text screening should be interpreted with caution, as it is very likely that the poor performance of full-text reported reflects this limitation. However, it should be noted that most of the other AI tools currently available, with the notable exception of A4SLR,^20^ do not have full-text screening capabilities. Third, our AIaS evaluated economic burden (cost and HCRU) from observational studies. Findings should not be generalized to other topics or disease areas because results may vary when deployed in different study designs or outcomes (eg, clinical SLRs that focus on randomized controlled trials).

Given the rapid development of AI technologies, it is reasonable to expect that new AI models will emerge in the medium to long term, with improved performance. For example, Lee and colleagues reported high performance of an agentic AI platform for all steps in SLR.^20^ In addition, the Cochrane AI Methods Group recently announced plans for a new study that will evaluate the quality of AI tools in updating 15 Cochrane reviews.^36^ These initiatives, along with the new RAISE framework, present a promising outlook and underscore that ongoing evaluation and adaptation are necessary to ensure that evidence synthesis methods continue to benefit from these advancements while maintaining methodological rigor.

## CONCLUSIONS

In essence, the use of AI in evidence synthesis must meet the same high standards of quality of traditional SLRs, and reviewers remain ultimately responsible for the quality and scientific rigor of their reviews. AI is rapidly evolving and providing exciting opportunities to accelerate literature reviews. Our AIaS showed that, at the current time, AI can support specific review tasks but still requires strong human supervision to maintain quality. To reduce risks when using AI in evidence synthesis, we suggest using the human-in-the-loop approach to maintain human oversight at each stage, including the initial decision of where AI should be used, what type of tool should be used, and how to validate AI decisions and outputs.

AI should be deployed in evidence synthesis only when its performance has been monitored by AI-literate human supervisors that can make an informed assessment of benefits and risks. Standardized quality assurance frameworks are needed to ensure compliance with responsible AI use, including RAISE recommendations and legal requirements. A framework for the validation and quality assessment of AI systems for evidence synthesis use remains a critical need.

### Disclosures

This study, as well as medical writing and editorial support for the manuscript, was sponsored by Astellas Pharma Inc. All stages of the manuscript preparation, including the review and approval process, were conducted with the aim of maintaining scientific integrity and transparency. Evidera Inc. receives fees for its consulting services from a range of companies across the healthcare sector.

## Supplementary Material

Online Supplementary Material
