# Supplementary material for: Evaluating AI Performance in Systematic Literature Reviews for HEOR: A Case Study
Source: J Health Econ Outcomes Res. 2026 Aug 7;13(2):46–54. doi: 10.36469/001c.165173 (PMC13453151; doi:10.36469/001c.165173)
Supplement: Supplementary file 1 [file jheor_2026_13_2_165173_357625.pdf]

Journal of Health Economics and Outcomes Research

## Online Supplementary Material

Evaluating AI Performance in Systematic Literature Reviews for HEOR: Methods and Recommendations. *JHEOR*. 2026;13(2):46-54. doi:10.36469/jheor.2026.165173

**Figure S1: Methods Used in the Traditional SLR**

**Table S1: Data Extraction Process Using Smart Tags**

**Table S2: Data Sources**

**Table S3: PICOS Eligibility Criteria**

**Table S4: AI Configuration**

**Table S5: Prompts Used for Full-Text and Data Extraction**

**Figure S2: Hierarchy of Prompts Designed for AI Data Extraction**

This supplementary material has been provided by the authors to give readers additional information about their work.

This is an open-access article distributed under the terms of the Creative Commons Attribution 4.0 International License (CCBY-4.0). View this license's legal deed at http://creativecommons.org/licenses/by/4.0 and legal code at http://creativecommons.org/licenses/by/4.0/legalcode for more information.

**Figure S1.** Data Extraction Process Using Smart Tags

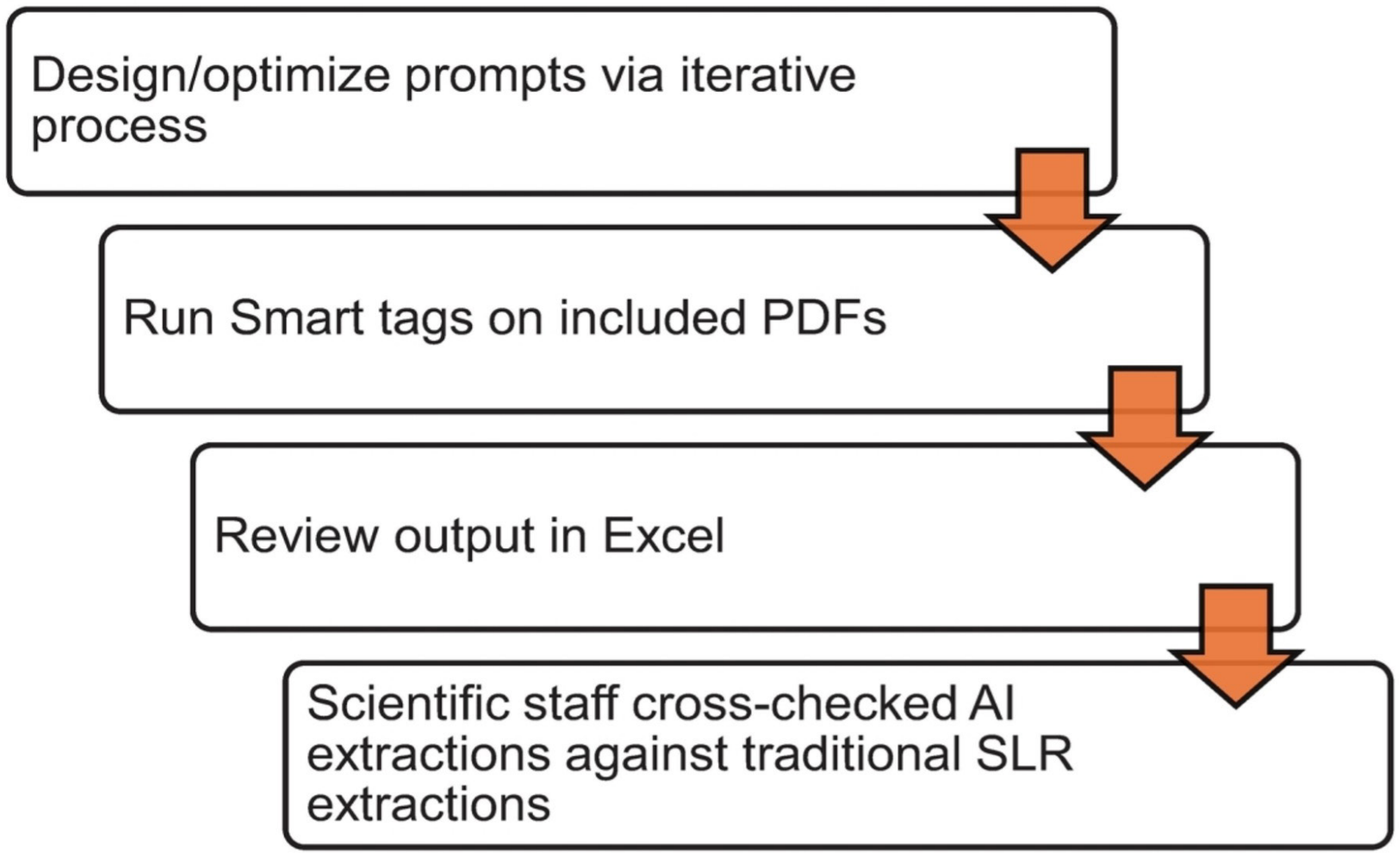


Abbreviations: AI, artificial intelligence; SLR, systematic literature review.

**Table S1.** Methods Used in the Traditional SLR

| SLR Task | Methods |
|---|---|
| Title/abstract screening | Double screening: each record was screened against the prespecified PICOS eligibility criteria by 2 independent reviewers. Any disagreements were resolved in consultation with a senior systematic reviewer. |
| Full-text screening | Records that passed the title/abstract screening were obtained as full-text, including all available supplementary materials.<br>Double screening: each full-text and supplementary materials were screened against the pre-specified PICOS eligibility criteria by 2 independent reviewers. Any disagreements were resolved in consultation with a senior systematic reviewer. |
| Data extraction and validation | Data were extracted by 1 reviewer and validated in full by an independent reviewer. Any disagreements were resolved in consultation with a senior systematic reviewer. |

Abbreviation: PICOS, population, interventions, comparisons, outcomes, and study design.

**Table S2.** Data Sources

| Types of Sources | Data Source via OvidSP |
|---|---|
| Literature databases | Embase<br>MEDLINE and MEDLINE In-Process<br>EconLit |
| Conference proceedings | Conference abstracts were searched via the searches in Embase. The in-house SLR conducted additional hand searches on the websites of the conferences listed below. The AI SLR included only conferences indexed in Embase (years indexed in Embase are shown)<br>AACR: 2020, 2021<br>AACR Pancreatic Cancer Special Conference: 2019-2023<br>ASCO: 2019-2023<br>ASCO-GI: 2021-2023<br>DDW: 2019-2023<br>ESMO: 2019-2024<br>ESMO World Congress on Gastrointestinal Cancer: 2019-2021, 2023<br>ESMO Japan: Unavailable in Embase<br>ESMO Asia: 2019, 2020, 2022, 2023<br>ISPOR EU: 2019-2023<br>ISPOR US: 2019-2023<br>ISPOR Asia Pacific: 2020 |

Abbreviations: AACR, American Association for Cancer Research; ASCO, American Society of Clinical Oncology; ASCO-GI, American Society of Clinical Oncology Gastrointestinal Cancers Symposium; DDW, Digestive Disease Week; ESMO, European Society for Medical Oncology; ISPOR, The Professional Society for Health Economics and Outcomes Research.
Note: Conferences not indexed in Embase were not searched separately, as NK AI does not have this capability. The inclusion of conference abstracts via hand searching was not considered when comparing results between AI-assisted and in-house SLRs.

**Table S3.** PICOS Eligibility Criteria

| Criteria | Include | Exclude |
|---|---|---|
| Population | Patients aged ≥18 years with metastatic pancreatic adenocarcinoma | • Diseases other than pancreatic adenocarcinoma<br>• Resectable or locally advanced, nonmetastatic disease<br>• Patients receiving maintenance therapy (ie, those with response or stable disease after 4-6 mo)<br>• Pediatric population<br>• Nonhuman population (in vitro, ex vivo, or animal studies) |
| Intervention/ comparators | Any approved or investigational therapy, placebo, standard of care, or no treatment | NA |
| Outcomes | • Total costs (direct + indirect)<br>• Direct costs<br>• Indirect costs (ie, productivity loss)<br>• Healthcare resource use (eg, hospitalization, outpatient visits, pharmacy use, procedures) | • Outcomes other than those listed (eg, epidemiology, pharmacokinetics, economic evaluations)<br>• Outcomes not separable for the population of interest |
| Study design | • Observational studies<br>• Cost-of-illness studies<br>• SLRs and narrative reviews | • Clinical trials<br>• Guidelines |
| Publication type | • Articles, including errata and retractions<br>• Conference abstracts | • Trial protocols<br>• Editorials, comments, notes, and letters |
| Language | English language | Full-text articles not published in English |
| Publication date | • Full-text articles: 2014-2024<br>• Conference abstracts: 2019-2024 | • Full-text articles published prior to 2014<br>• Conference proceedings published prior to 2019 |

Abbreviations: NA, not applicable; PICOS, population, interventions, comparisons, outcomes, and study design; SLR, systematic literature review.

**Table S4.** AI Configuration

| Characteristics of the AI Screening Model (Robot Screener)[a] | Characteristics of Smart Tag Recommendations[a] |
|---|---|
| • Gradient-boosted decision tree ensemble. Its hyperparameters, particularly around model complexity (number of trees, tree depth) are optimized using a cross-validation grid search.<br>• The model produces posterior probabilities and is optimized on logistic loss.<br>• Synthetic Minority Oversampling Technique (SMOTE) oversampling is employed as a correction to highly imbalanced classes frequently seen in screening.<br>• The **Robot Screener** is a machine-learning model that is trained on the records' bibliographic data (ie, time since publication, page count, key words and descriptors), abstract content (ie, n-gram language model, OpenAI text embedding such as text-embedding-(ada-002) to measure how text strings are related), and citation counts (ie, number of citing publications, number of supporting citation statements, number of contrasting citation statements).<br>• If any of these fields are missing from an individual record, it is imputed as if the record is approximately typical to other records in the nest. | • Smart Tag Recommendations is a feature specific to NK.<br>• Smart tags is a content extraction large language model (LLM) that uses Generative Pre-trained Transformer 4 (GPT-4).<br>• This feature identifies the most relevant text in the PDF based on human-generated prompts and returns plain text via a type of search called "fuzzy query," which searches for the most accurate answer to the prompt by measuring edit distance (also known as Levenshtein distance) in the text. |

[a]Further details can be found at: https://wiki.nested-knowledge.com/doku.php?id=wiki:support:ai_disclosure

**Table S5.** Prompts Used for Full-Text and Data Extraction[a]

| **PICOS Criteria** | **Prompt** | **Aliases Used**[b] | |
|---|---|---|---|
| Population | Study population | What population was studied? | – |
| | Disease | Did this study include patients with pancreatic cancer? | pancreatic ductal adenocarcinoma<br>pancreatic adenocarcinoma<br>ductal carcinoma of pancreas<br>carcinoma of pancreas<br>ductal carcinoma of the pancreas<br>pancreas ADK<br>squamous cell carcinoma of the pancreas<br>adenosquamous carcinoma of the pancreas<br>colloid carcinoma of the pancreas |
| | Disease stage | Did patients in this study have metastatic cancer? | advanced<br>metastatic<br>metastases |
| Intervention/ comparators | Treatment | What treatment/s were administered to patients? | – |
| | Previous treatment(s) | Did patients receive any treatments prior to enrollment in the study? | – |
| | Line of therapy | What was the line of therapy patients received within the study? | – |
| Outcomes | Outpatient costs | What were the costs associated with outpatient visits? | primary care<br>physician<br>doctor<br>laboratory<br>technician<br>specialist |
| | Indirect costs | What were the data associated with indirect costs of care? | sick days<br>absenteeism<br>presenteeism<br>work productivity<br>missed work<br>missed school<br>travel<br>caregiver |
| | Length of stay | What was the duration of time in which patients had a hospital stay? | length of stay<br>duration of hospitalization<br>LOS<br>hospital stay |
| | Emergency (%) | What is the proportion of patients who had an emergency department or urgent care visit? | emergency room<br>emergency department<br>urgent care |
| | Emergency (No.) | What was the mean or median number and standard deviation or range of emergency department or urgent care visits? | emergency room<br>emergency department<br>urgent care |
| | Outpatient (%) | What is the proportion of patients with outpatient visits (overall or by individual type of outpatient visit (physician, doctor, primary care, specialist, laboratory, etc)? | primary care<br>physician<br>doctor<br>laboratory<br>technician<br>specialist |
| | Outpatient (No.) | What was the mean or median number and standard deviation or range of outpatient visits (overall or by individual type of outpatient visit (physician, doctor, primary care, specialist, laboratory, etc)? | primary care<br>physician<br>doctor<br>laboratory<br>technician<br>specialist |

**Table S5.** Prompts Used for Full-Text and Data Extraction[a]

| PICOS Criteria | Prompt | Aliases Used[b] | |
|---|---|---|---|
| Study design | Study design–general | What is the study design? | – |
| | Study design–prospective | Is this a prospective observational study? | – |
| | Study design–retrospective | Is this a retrospective observational study? | – |
| | Study design–clinical trial | Is this a clinical trial? | – |
| Clinical trial | Single arm trial | Is this a clinical trial with only one treatment group? | – |
| | RCT | Were patients randomized to their treatment groups? | – |
| | Non-RCT | Was this a clinical trial with multiple treatment arms but without randomization of patients to treatment groups? | – |

Abbreviations: ADK, ***; LOS, length of stay; RCT, randomized controlled trial.
[a]A representative selection of prompts is provided. The full library of prompts is available from the authors upon reasonable request.
[b]An alias is an alternative name for the same concept to aid the model.

**Figure S2.** Hierarchy of Prompts Designed for AI Data Extraction[a]

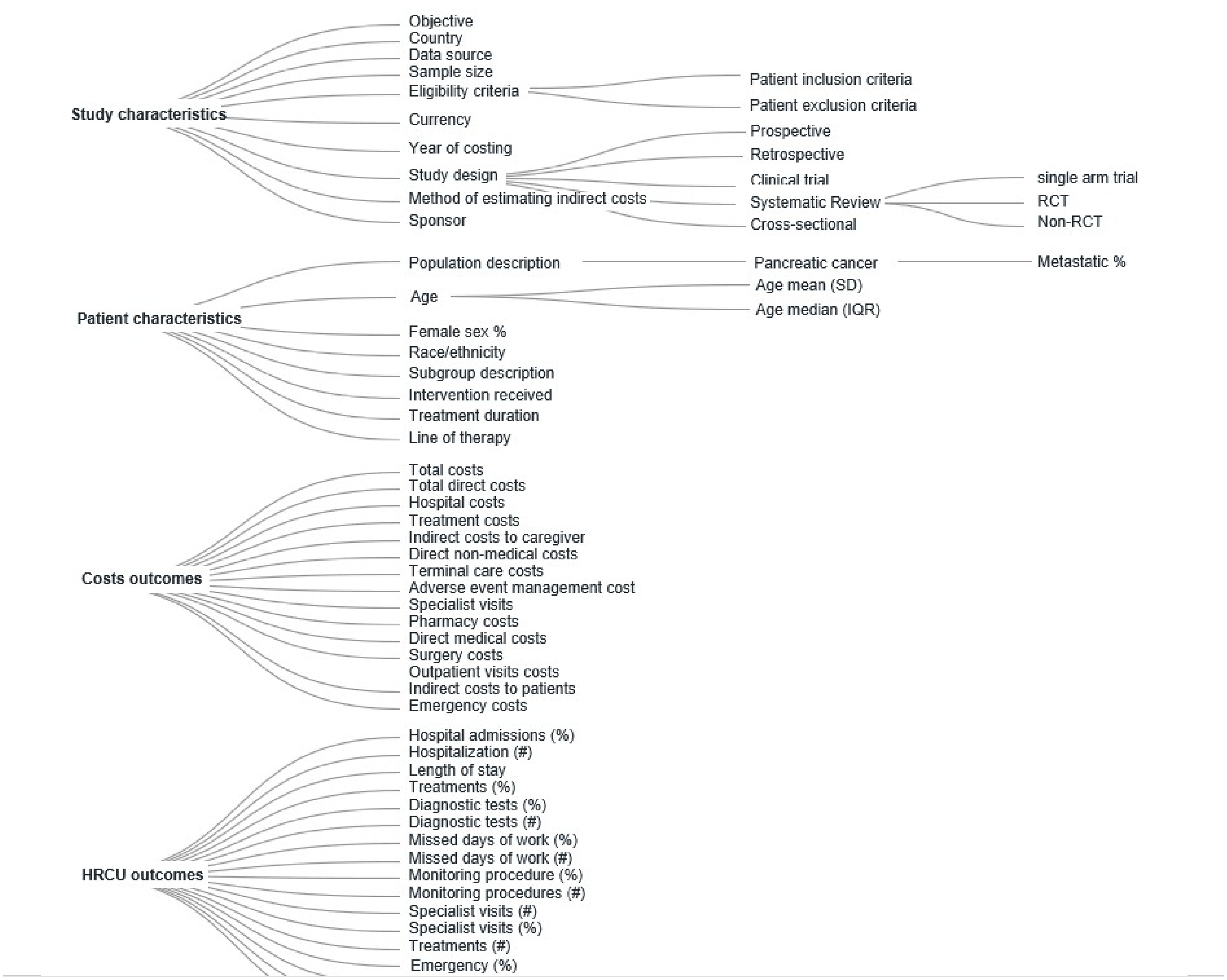


[a]A representative selection is provided. The full list of prompts is available from the authors upon reasonable request.
